# Taking a holistic view of PEST‐containing nuclear protein (PCNP) in cancer biology

**DOI:** 10.1002/cam4.2465

**Published:** 2019-09-05

**Authors:** Attia Afzal, Muhammad Sarfraz, Guang‐Lei Li, Shao‐Ping Ji, Shao‐Feng Duan, Nazeer Hussain Khan, Dong‐Dong Wu, Xin‐Ying Ji

**Affiliations:** ^1^ Henan International Joint Laboratory for Nuclear Protein Regulation Henan University Kaifeng China; ^2^ Faculty of Pharmacy The University of Lahore Lahore Pakistan; ^3^ Muncipal Key Laboratory of Cell Signal Transduction Henan Provincial Engineering Centre for Tumor Molecular Medicine Henan University Kaifeng China; ^4^ Institute for Innovative Drug Design and Evaluation School of Pharmacy Henan University Kaifeng China; ^5^ School of Basic Medical Sciences Henan University College of Medicine Kaifeng China; ^6^ Kaifeng Key Laboratory of Infection and Biological Safety (KLIBS) Henan University College of Medicine Kaifeng China

**Keywords:** apoptosis, MAPK, PCNP, PEST, STATs, tumor, Ubiquitin

## Abstract

Polypeptide sequences enriched with proline (P), glutamic acid (E), aspartic acid (D) and serine (S)/ threonine (T) (PEST) have been reported to be the most abundant and frequently distributed at the cellular level. There is growing evidence that PEST sequences act as proteolytic recognition signals for degradation of residual proteins which is critical for activation or deactivation of regulatory proteins involved in cellular signaling pathways of cell growth, differentiation, stress responses and physiological death. A PEST containing nuclear protein (PCNP) was demonstrated as a tumor suppressor in a neuroblastoma cancer model and tumor promoter in lung adenocarcinoma cancer model. Its unique properties like ubiquitination by NIRF, co‐localization with NIRF in nucleus and tumor progression attract the attention of researchers. PCNP was reported to be ubiquitinated by ring finger protein NIRF in E3 ligase manner and as modulator of MAPK and PI3K/AKT/mTOR signaling pathways. In this review, we summarize PCNP linked DNA damage response, Post translational modifications, and transportation to address initiation, prognosis, and resistance of tumor cells in terms of cell cycle regulation, transcription and apoptosis. Hence, we demonstrate PCNP as a novel target in cancer diagnosis and treatment.

## Graphical abstract

Involvement of PCNP in cell cycle regulation, transcription and apoptosis is demonstrated in this review by integrating online available scientific data about PCNP or related nuclear proteins. A very unique intermediate role of PCNP in cancer immunity is also proposed in this review.

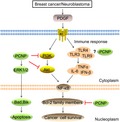

## 1 INTRODUCTION

PEST containing nuclear protein (PCNP) is a novel finger protein involved in the cell cycle regulatory process.^1^ PEST is a polypeptide lined with at least one proline (P), one glutamic acid (E), aspartic acid (D) and one serine (S)/ threonine (T). It was proposed in 1986 that PEST‐linked proteins have rapid destruction due to associated PEST regions.^2^, ^3^, ^4^ PEST containing proteins are abundant and are involved in nutrient regulation of cellular metabolism and physiology,^5^ nucleo‐cytoplasmic transport,^3^ cell cycle regulation,^1^ cyclic nucleotide signaling pathways^6^ and stability of nuclear protein.^7^ PEST containing proteins interfere at the molecular level with the ubiquitin proteasome pathway,^4^ glycosylation of the nuclear pore^3^, ^4^ and hexosamine biosynthetic pathway (HBP).^7^ PCNP mediates the proliferation, migration, and invasion of human neuroblastoma and lung adenocarcinoma cells, probably via ubiquitination. PCNP may act as a transcriptional factor, cell cycle regulatory protein and tumor regulating nuclear protein.^1^, ^8^, ^9^, ^10^

## 2 PEST SEQUENCE

Chemically, PEST sequences are single or multiple stretches of acidic and hydroxylated amino acids usually present as carboxy‐terminal extensions of residual proteins. PEST motifs are greater than or equal to 12 residues in length, in case; 10‐50 residues long. PEST motifs contain unusually high densities of prolines, the acidic residues glutamate and aspartate, and the hydroxylated amino acids serine and threonine. It may or may not be winged with lysine (K), arginine (R) or histidine (H) residues. Though PEST sequences share no primary sequence identity with each other, however, they share overall character—protein sequences. Among different residues of PEST motif, an acidic motif enriched with serines and threonines was found responsible for Ste6p ubiquitination and rapid turnover.^11^ Lysine in PEST sequence appears to serve as the acceptor site for ubiquitin attachment.^12^ Prolines of PEST sequence play a role in specifying the construction of the multi‐ubiquitin chain. It was observed that the rate of ubiquitination reflects the size of PEST motif of the substrate; larger and more negatively charged sequences may provide better substrates for ubiquitination.

The presence of PEST motif on proteins can be detected using PEST‐FIND. It is a computer aided program^2^ which could positively detect the PEST motifs especially enrich with at least one P, bounded with basic residues (K, R, or H), minimum one acidic residue (D or E), and one hydroxylated amino acid (S or T). PEST motifs are frequently distributed among cellular proteins especially immunogenic proteins, metabolic enzymes, transcription factors, protein kinases, protein phosphatases and cyclins.^2^ Most of the nuclear proteins; Myc, Fos, Jun, p53, etc are enriched with P, E, S and T. PCNP and another PEST containing nuclear protein, NIRF (Np95/ICBP90‐like RING finger protein) were also underlined by PEST‐FIND with a positive value (5.89 and 10.89).^3^ These motifs become attached to the COOH terminus of residual protein, for example protein kinases, and act as signal for constitutive proteolytic activity. However these motifs initiate proteolytic activity in response to external stimuli as light and phosphorylation stimulate the ubiquitin‐26S protease pathway.^13^, ^14^, ^15^ PEST regions are transplantable proteolytic signals.^15^

## 3 UBIQUITINATED PCNP REGULATES TUMOR CELL CYCLE

**Figure 1:**
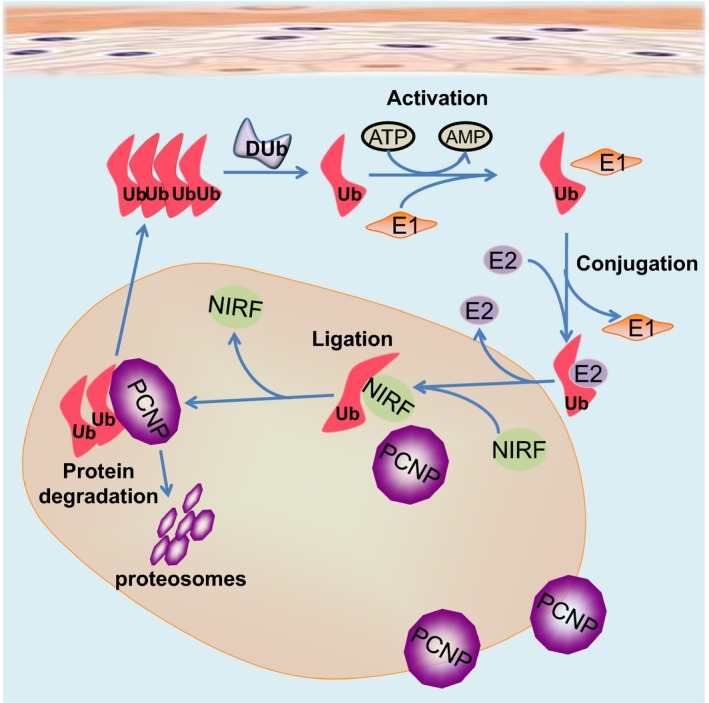
Ubiquitination of PEST containing nuclear protein (PEST) by NIRF (Np95/ICBP90‐like RING finger protein) in the nuclear core of the cell: Ubiquitin‐like domain in the N‐terminus and a RING finger motif in the C‐terminus of NIRF confirm the ubiquitin ligase function of NIRF. PCNP acts as a substrate for NIRF mediated proteasome activity. Sub cellular nuclear colocalization of PCNP and NIRF was visualized using Fluorescent microscope by transfecting fusion construct in COS‐7 or HEK‐293 cells. Binding of PCNP and NIRF was studied using GST pull‐down assay

Ubiquitin conjugating and ligase enzymes regulate the life cycle of cancer cells which is manifested by elevated levels of these enzymes in cancer microenvironment and serve as tumor biomarker.^16^ Ubiquitin, a small 76‐residue protein, has long been known as a regulator of protein degradation in the cell cytoplasm. Ubiquitin is first bound and activated by the E1 ubiquitin activating enzyme in an ATP‐dependent manner. It is subsequently transferred to the E2 ubiquitin conjugating enzyme, before the E3 ubiquitin ligase enzyme specifically binds the substrate protein to mediate covalent attachment of ubiquitin with a selected lysyl residue, which targets the substrate protein for proteasomal degradation.^17^, ^18^ Ubiquitination controls the expression of cell cycle regulating cyclin proteins. The mammalian cell cycle is a strictly regulated process controlled by the oscillating activities of cyclin‐dependent kinases (Cdk) in normal conditions. The prototype cyclins interact with Cdk1 or Cdk2 in a cell cycle dependent manner to mediate cell cycle regulatory processes such as DNA replication and mitosis.^19^ Among the PEST containing cyclins, cyclin E which is ubiquitinated by E3 ubiquitin ligase,^20^ is activated in late G1 phase to promote S‐phase entry and DNA replication.^19^ Cyclin E‐Cdk2 and (later) cyclin A‐Cdk2 are the two main Cdk complexes in S‐phase which mediate the initiation of DNA and centrosome duplication. NIRF interacts with a variety of proteins involved in diverse cellular processes, for example, cell cycle‐promoting factors (cyclins: cyclin E1 and cyclin D1 and Cdks) and cell cycle‐inhibitory factors (pRB and p53) which are core components of the cell cycle machinery. NIRF induced G1 arrest by ubiquitinating the cyclins D1 and E1.^21^ Expression of nuclear protein NIRF was found to be higher in the proliferating phase but significantly lower in the G0‐G1 phase in normal TIG‐7 and WI‐38 cells, while being consistently higher in tumoral HT‐1080 and HepG2 cells in both phases. The NIRF gene is positioned at a point responsible for chromosomal DNA amplification in various types of tumors, suggesting that NIRF is involved in cell cycle regulation and tumorigenesis in certain types of human tumors.^1^, ^22^, ^23^ NIRF was observed as a partner interacting with PCNP in yeast two‐hybrid screening.^1^ NIRF, acting as ubiquitin ligase, was involved in the ubiquitination of PCNP in HEK‐293 and COS cells, in vitro and in vivo, as illustrated in Figure 1. Hence, it may be concluded that the short‐lived regulatory protein PCNP communicates with NIRF in the signaling pathway of cell cycle regulation and/or genome stability.

It is further inferred from the above discussion that as cell cycle‐promoting factors cyclin E1 and cyclin D1 as well as cell cycle‐inhibitory proteins are ubiquitinated by E3 ligase and NIRF, PCNP is also one of such cell cycle regulatory proteins which is ubiquitinated by NIRF. Like p53 and pRB, PCNP acted as tumor suppressor cell cycle regulatory protein in neuroblastoma, while like cyclin E and cyclin D, it acted as tumor promoting cell cycle regulatory protein in lung adenocarcinoma, in vitro and in vivo.^10^ Similarly, higher concentrations of PCNP would not be expected during the proliferation and transition phase of cell cycle in response to high expression of NIRF during the resting and proliferating phase of tumoral cells.

## 4 PCNP MEDIATES CHROMATIN MEDIATED TRANSCRIPTION

Post translational modifications (PTMs) in proteins, responsible for initiation of cellular responses through transcriptional activation or repression of distinct target genes, are induced by numerous carcinogens (like ultraviolet radiation). As we discussed somewhere that oxidative stress causes thiolytic cleavage of cellular proteins initiating mitogen‐activated protein kinases (MAPK) and nuclear factor‐kappa B (NF‐*κ*B) pathway which disturb the cellular integrity.^24^ The PTMs like phosphorylation and methylation markedly influence the expression of their target genes.^25^ An oversupply or overactivity of transcription factors via nuclear proteins is responsible for unrestrained growth and metastatic behavior of all human cancers.^26^ The transcription factors are short lived, rich in PEST sequences and are controlled by proteolysis, mostly via ubiquitin‐mediated degradation. The transcription factors with oncogenic potential are steroid receptors, resident nuclear proteins and latent cytoplasmic factors. For example, induction of apoptosis in multiple myeloma and Hodgkin's disease cells was observed when dominant negative NF‐*κ*B subunits were introduced in these cells. Cytoplasmic activation of NF‐*κ*B is introduced by a complex cellular mechanism, that is, interleukin‐1 (IL‐1) or tumor necrosis factor‐*α* (TNF‐*α*) like proteins attach with cytokine receptors and gain entry into the cell where they stimulate destruction of the inhibitor of *κ*B (I*κ*B) via phosphorylation and proteolysis releasing protein p65. A heterodimer of p65 and p50 is formed as an active transcription factor which enters into the nucleus and releases NF‐*κ*B. The latent transcription factors, for example, signal transducers and activators of transcription (STATs) most often enhance transcription by interacting with other transcription factors on chromatin otherwise until transferred to the nucleus.^27^ Activation of cell surface receptors leads to the activation of associated Janus‐ or TYK2‐kinases (designated as JAK), and JAK activates STAT3/5 predominantly via tyrosine phosphorylation while inhibiting STAT3/5 by tyrosine phosphatases. The recruitment and tyrosine phosphorylation of STAT molecules is a process which occurs in the cytoplasm.^28^, ^29^ The pSTAT3 accumulates in nucleus and binds to DNA leading to transcription and angiogenesis. Dysregulated transcription is considered to be the molecular basis for limitless replication potential and avoidance of apoptosis moving towards tumorigenesis. High levels of STAT3/5 were observed in over expressed PCNP adenocarcinoma cells. One may confer here that higher expression of STATs and PCNP with associated higher proliferation, migration, and invasion as compared to control is due to the interaction of PCNP and STATs enhancing the transcription.

Transcription is regulated by histone post translational modification (PTM); chromatin remodeling and other such mechanisms.^30^ In fact, high level of histone H3K4/79 methylation is a prerequisite for initiation and elongation of transcription. The PHD domain in chromatin remodeling factors (e.g. NURF) and histone modification complexes specifically recognizes modified products which are responsible for initiation of transcription.^31^ In case of NIRF, being a resident of the nucleus with PHD finger domain, NIRF is responsible for ubiquitination and phosphorylation which is an important PTM of chromatin in transcriptional activation.^22^ Post translational modification of tumor suppressor protein p53 is disrupted by overexpression of mouse double minute 2 (MDM2) ubiquitin ligase, a phenomenon observed in many tumor types. MDM2 is a negative regulator of p53 and act as oncogene.^25^, ^32^, ^33^ Nuclear factor E2‐related factor‐2 (Nrf2), is an essential transcription factor, which is ubiquitously controlled by ubiquitin ligase E3, affects down regulation of pro‐inflammatory cytokines.^34^ Similar to p53 and Nrf2, PCNP being a substrate for NIRF undergoes PTM which may have an important role in the activation of transcription factor, that is, stimulation of the STATs signaling pathway via interaction of STATs with PCNP on chromatin. Furthermore, a high PEST score and presence of CK2‐phosphorylation sites are reminiscent of key molecules governing cell cycle progression and transcriptional regulation.^8^, ^35^ Moreover the phosphorylation potential increases with PEST score. Hence, with high PEST level, one may expect that ubiquitinated PCNP is one of the important modified complexes of the chromatin mediated transcriptional pathway. This post translational modification of PCNP is regulated by NIRF having a PHD ring finger domain which together with/ without STATs may involve in initiation and regulation of chromatin mediated transcription.

## 5 PCNP REGULATES APOPTOTIC INDEX VIA TRIF DEPENDENT IMMUNITY AND PI3K SIGNALING PATHWAY

**Figure 2:**
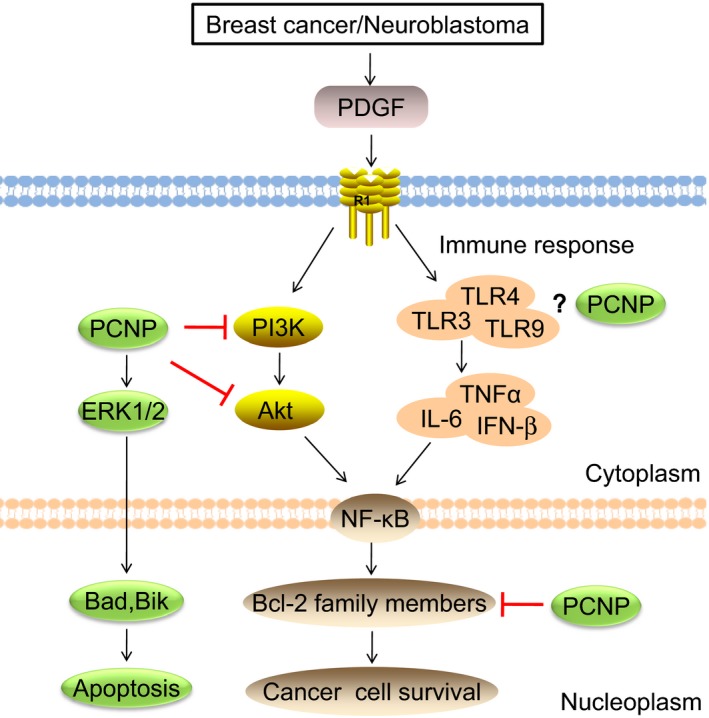
Platelet‐derived growth factors from breast cancer or neuroblastoma stimulate the platelet‐derived growth factor (PDGF) receptors and initiate immune and tumorigenesis response. Both pathways finally produce NF‐*κ*B (nuclear factor kappa‐light‐chain‐enhancer of activated B cells) which in turn disturbs the apoptotic balance. PCNP (PEST containing nuclear protein) inhibits the tumorigenesis pathway (PI3K, Akt) and increases the apoptotic balance. Hence, it increases the apoptosis

The cytokines interleukins IL‐1*β* and tumor necrosis factor alpha (TNF*α*) mediate innate immunity via two distinct pathways MyD88 and Toll/IL‐1R domain‐containing adaptor protein inducing IFN‐*β* (TRIF). Ubiquitin regulates the MyD88‐ and TRIF‐dependent pathways.^36^ For example, the MyD88‐dependent pathway involves in the early phase NF‐*κ*B activation while the TRIF‐dependent pathway involves in the late phase NF‐*κ*B activation.^37^, ^38^ TRIF induces Toll‐like receptors (TLRs) among which TLR‐4 was ubiquitously expressed by epithelial ovarian cancer cell lines.^39^ TLRs together with the downstream modulator TRAF6 regulate the expression of cytokines. Specifically talking about breast tumors, inflammation within the tumor microenvironment is related to increased invasiveness and poor prognosis which in turn is related to the expression of the proinflammatory cytokines IL‐6, TNF*α* and IL‐1*β*.^40^ These proinflammatory cytokines are regulated by TRAF6 induced by TLRs. TRAF6 is targeted by ubiquitin ligase to prevent dysregulated inflammatory response triggered by TLRs.^41^ Furthermore, clinical studies demonstrated significantly higher levels of circulating IL‐6 in estrogen receptor negative (ER‐) cell lines as compared to ER+ cell lines (ex vivo) which were associated with the poor prognosis of the disease.^40^, ^42^ In case of IL‐1*β*, it has been linked to migration, increased expression of cyclooxygenase‐2 (Cox‐2) and hypoxia inducible factor‐1 alpha (HIF‐1*α*) leading to angiogenesis and metastasis in the breast cancer cell line, MCF‐7.^43^ TRIF‐dependent immunity mediated by TLRs was trunked by ubiquitination of TRIF and suppressed the activity of NF‐*κ*B.^44^, ^45^ As shown in Figure 2, a ubiquitously regulated nuclear protein PCNP increased the phosphorylation of ERK1/2, JNK, and p38 protein kinases mediating the MAPK pathway to promote apoptosis in human neuroblastoma cells.^8^ Moreover, PCNP over expressed cancer cells decreased phosphorylation of PI3K (Tyr458/ Tyr199), AKT (Ser473), and mTOR (Ser2448). ERK1/2 influences the expression and/or activity of Bcl‐2 family members. PI3K and AKT activate NF‐*κ*B which generates antiapoptotic proteins improving the survival of cancer cell.^8^ PCNP inhibited the production of transcription factor NF‐*κ*B by inhibiting the PI3K/Akt signaling pathway. MyD88 and TLR4s are upstream prerequisite molecules for MAPK and PI3K/Akt activation.^46^ This pathway culminates in the phosphorylation of the Bcl‐2 family members, thereby suppressing apoptosis and promoting cell survival.^47^ PCNP overexpression remarkably increased the apoptotic index in neuroblastoma cell lines, protein expressions of cleaved caspase 3, 8, 9, as well as Bax/Bcl‐2 and Bad/ Bcl‐xl ratios. The higher Bax/Bcl‐2 and Bad/ Bcl‐xl ratios in over expressed PCNP neuroblastoma cells as compared to control, are due to stimulation of TRIF‐ or MyD88‐dependent immunity, remains unknown.

On the other hand, PCNP mediates the proliferation, migration, and invasion of human lung adenocarcinoma cells via the pSTAT3/5 and PI3K/Akt/mTOR signaling pathways.^10^ Mitogen‐activated protein kinases and downstream regulators like PI3K/Akt/mTOR are stimulated by growth factors and cytokines. The increased regulation of PI3K generates 3′‐phosphorylated lipid products, and activates PKB/Akt leading to antiapoptotic response.^10^, ^48^ Activation of receptor tyrosine kinase (RTK) increases the phosphorylation of STAT3 and its nuclear translocation leading to increased transcription. So it can be inferred here that stimulation of the PI3K/Akt/mTOR signaling pathways not only promoted angiogenesis in adenocarcinoma cell lines via STAT3/5 phosphorylation but also inhibited the apoptotic activity of cells leading to angiogenesis. As we discussed earlier that STATs are activated by phosphorylation triggered by growth factor receptors stimulation, this activating signal can be considered to be responsible for inactivation of the (MyD88‐/TRIF‐dependent) immune system which is triggered by cytokines.

## 6 UBIQUITINATION IS RESPONSIBLE FOR TRANSLOCATION OF PCNP

Protein complexes are constantly being formed and resolved in the dynamic architecture of eukaryotic cells to execute cellular signaling, and proteins are shuttling between different subcellular localizations to accomplish biological processes.^49^ Subcellular localization of tumor suppressor protein is important for apoptotic activity. The mechanism for intracellular localization and respective cellular response due to subcellular translocation of some of nuclear proteins have been summarized in Table 1. Mitochondrial mediated apoptosis is either intrinsic (activated by toxins, drugs, viral infections, free radicals, hypoxia, hyperthermia, calcium flux, loss of growth factors, cytokines or hormones, and elimination of apoptotic suppression and resulting in activation of caspase 9) or extrinsic (activated by active cell‐surface death receptors resulting in activation of caspase 8 and 10) which are converged into a common downstream pathway releasing different effectors on the way like Bax and Bid which are translocated to different parts of the cell.^50^, ^51^ Tumor suppressor proteins, like p53, reside in nuclear pore as well as in the cytoplasm. The important functions of p53 such as cell cycle regulation, DNA damage repair, and apoptosis, can be inhibited by cellular compartmentalization of p53.^52^ Cytoplasmic p53 was sequestered by antiapoptotic Bcl‐xl. However, nuclear p53 caused activation of PUMA (proapoptotic activator) which released cytoplasmic p53 from Bcl‐xl. This free cytoplasmic p53 stimulated the effector proteins leading to mitochondrial outer membrane permeabilization which in turn triggered the “executioner” caspases 3, 6 and 7.^53^, ^54^ Similarly, Livin was introduced as a nuclear protein with remarkable apoptotic activity. However, it was found that translocation of the truncated Livin to Golgi apparatus is prerequisite for its apoptotic activity. It was demonstrated by biochemical analysis at increasing detergent gradient and increasing salt gradient, as well as by fluorescence analysis that Livin concentrates in an asymmetric peri‐nuclear structure colocalized almost completely with the Golgi marker N‐acetylglucosaminyl transferase (NAG).^55^ The nuclear translocation of quaking gene isoform‐7 (QKI‐7) induced by heterodimerization suppressed apoptosis.^56^ As far as PCNP is concerned, PCNP and NIRF are colocalized in the nucleus as observed in vitro and in vivo. The plasmid EGFP‐PCNP, transfected into COS‐7 cells, was exclusively localized to the nucleus, somewhat homogeneously outside the nucleolus, avoiding nucleoli when observed under a fluorescence microscope for sub cellular localization.^1^ It may induce apoptotic activity by directly acting on the DNA/ nucleus of the cancer cell; if it is true then the mechanism of caspase 8 released by over expressed PCNP in neuroblastoma is a question. The insights into release of PCNP from the nucleus to the cytosol would be helpful to understand the mechanism of PCNP‐mediated apoptosis. Localization of proteins under stress is an important factor to evoke tumorigenic or tumor suppressive response. Tumor suppressor p53 was translocated to the mitochondria or nucleus due to redox potential produced by irradiation of UV‐A or UV‐B respectively and triggered either intrinsic apoptosis or direct DNA damage based apoptosis depending upon their respective accumulation.^57^ Redox potential was responsible for replacement of protein and initiation of apoptosis. In another study, Mutant p53 lost its transcriptional activity when accumulated in the cytoplasm of human hepatoma cells. Geranylgeranoic acid reversed this process by transporting the mutant p53 via importin α/β from the cytoplasm to the nucleus and up regulated PUMA gene expression in human hepatoma cells leading to cell death.^58^

**Table 1:** Translocation of nuclear proteins

Protein	Transport Initiated by	Transporters	Response	Reference
FoxO	Reactive oxygen species (ROS) and cysteine oxidation	Heterodimerization with the transportin‐1 (TNPO1) via an intermolecular disulfide bond	Nuclear localization of transcription factor and cell cycle arrest	73
cFos	Complex and conformation‐dependent	A combination of two NLSs	Nuclear localization of protein and transcription	74
Protein‐tyrosine phosphatase TCPTP (TC45)	Passive diffusion due to hyperosmotic stress	TC45‐NLS binds directly to importin *β*1, importin *α* and unidentified 116‐kDa protein	Cytoplasmic accumulation and regulates tyrosine phosphorylation‐dependent signal transduction events	75,76
Pak5	NLS in the extreme N terminus (residues 5‐10)	LMB‐sensitive Crm1‐dependent Pathway	Cytoplasmic translocation is important to phosphorylate bad protein and protect against apoptotic stimuli	77,78

Since key oncogenes and tumor suppressor function in the nucleus and have nuclear localization sequences (NLSs) and nuclear excretion sequences (NESs), unbalanced nucleo‐cytoplasmic shuttling of these factors is correlated with tumorigenesis**.** These are recognized by transport receptors termed as karyopherins, importins (a and b transportin, snurportin, etc), or exportins (Crm1/XPO/exportin1, etc).^59^, ^60^ Posttranslational modification is one of the signaling pathways involved in the nuclear transport regulation. Nuclear transport of NF‐*κ*B, p27 and p53 is regulated by posttranslational modifications such as phosphorylation of I*κ*B triggered nuclear import of NF‐*κ*B,^61^ phosphorylation and degradation of p27 down‐regulated Crm1 mediated export of p27,^62^ tributyrin mediated inhibition of nuclear exporter Crm1 restored normal functions of p53.^52^ PTEN monoubiquitination at Lys13 or Lys289 by NEDD4 increased its nuclear localization, whereas polyubiquitination by NEDD4 promoted its cytoplasmic degradation.^63^, ^64^ In response to DNA damaging signal, PTEN was monoubiquitinated by ATM serine/threonine kinase at serine 113, promoting PTEN nuclear translocation. PTEN nuclear translocation induced autophagy through activation of the p‐JUN‐SESN2‐AMPK Pathway.^65^ A ring finger protein (RNF144A) was up regulated in response to DNA damage and RNF144A induced ubiquitination of DNA‐PKcs in vitro and in vivo.^66^ Monoubiquitination and SUMOylation seem to enable nuclear export of p53.^67^

Once the tumor suppressor protein is localized at its active site, it generates antitumor response by releasing apoptotic proteins, promoting their subcellular localization and downstream stimulation of caspase tree hence apoptosis. Not only the subcellular localization of proteins, but the localization of effectors and executioners in response of apoptotic stimuli is also important. Post translational modifications like dimerization, phosphorylation, proteolysis and transcription also regulate BH3‐only proteins and are responsible for their translocation.^68^, ^69^ The cytosolic levels and subcellular localization of these apoptotic Bcl‐2 family proteins ultimately determine the commitment of a cell to the apoptotic program and induction of the programmed cell death cascade.^50^ Antiapoptotic members like Bcl‐2, Bcl‐xl reside in the outer mitochondrial membrane, endoplasmic reticulum membrane and nuclear envelope while proapoptotic members like active‐Bax reside in the cytosol. Upon stimulation, these proteins accumulate in the outer mitochondrial membrane and induce its permeabilization. It was observed that apoptosis was suppressed by humanin peptides via inhibition of Bax translocation to mitochondrial membranes.^70^

Overexpressed PCNP remarkably induced apoptosis via the mitochondrial mediated pathway which is evident with higher apoptotic index (Bax/Bcl‐2 and Bad/Bcl‐xl ratio), and protein expressions of cleaved caspase 3, 8 and 9 as compared to control.^8^ PCNP, being a nuclear protein, may be assumed to evoke the intrinsic pathway of apoptosis by phosphorylation of caspase 9. Although PCNP mediates this cascade via apoptotic proteins, PCNP mediates apoptosis directly from nucleus or after localization to cytosol, remains unveiled. If it resides in the nucleus and initiates apoptosis by direct DNA targeting, how are the effectors transported to the mitochondria for mitochondrial membrane localization, permeabilization and cell lysis? If PCNP is assumed to be translocated to cytosol like p53 and stimulates the effector proteins, what is the transport mechanism of PCNP? Similar is the case when in the neuroblastoma anticancer study, phosphorylation of caspase 8 was observed in over expressed PCNP in tumor cells by western blot which is the executioner of the extrinsic apoptosis pathway mediated by cell membrane death receptors.^51^ Caspase 8 from extrinsic pathway and Caspase 9 from intrinsic merge in a single downstream pathway via Caspase 3. These executers release the apoptotic activator Bid during downstream modulation which connects the both apoptotic cascades.^51^ In the study mentioned above, the effect of PCNP on the release of Bid was not observed.

## 7 CONCLUDING REMARKS

Tumor is induced by oncogenes and is expressed as higher levels of different cytokines which are propagated by number of signaling pathways and disrupt cell cycle regulation, compromise the innate immunity of the cell and alternate the cellular microenvironment as discussed and analyzed previously in our review report.^24^ Overexpression of cytokines is responsible for activation of transcription factors which are regulated by ubiquitination. An oversupply or overactivity of transcription factors via nuclear proteins is responsible for unrestrained growth and metastatic behavior of all human cancers.^26^ Ubiquitination not only regulates the protein substrate levels, but is also responsible for the localization and activity of key protein kinases in many signaling pathways, such as STATs, NF‐*κ*B and PI3K/Akt.^71^ To lineate the activity and function of a subset of proteins, including tumor suppressor and tumor promoter proteins, is critical for our understanding of tumor development, progression and sensitivity to drug treatment. PEST containing proteins are easy targets for ubiquitination. Among PEST containing nuclear proteins, PCNP is a short‐lived novel oncogene, ubiquitination of which serves as a novel chemotherapeutic target. The distinct properties of PCNP, like PEST motif and ubiquitination relate it with PTMs and cell cycle regulation but there are many unresolved questions which should be resolved, for example, (a) Although PCNP has good potential for phosphorylation, what are the factors responsible for and what kind of cellular responses are generated by this PTM?; (b) PCNP is ubiquitinated by NIRF in vitro and in vivo, (b1) what is the mechanism underlying the initiation/stimulation of PCNP ubiquitination under stress conditions?, (b2) whether ubiquitinated PCNP is tranclocated from nucleus to cytoplasm or not?; (c) The PTMs are responsible for translocation of BH3‐only apoptotic proteins; either the apoptotic index is disturbed by translocation of PCNP to cytoplasm or translocation of BH3‐only proteins via ubiquitination in response to DNA damage is responsible for this? (d) Over expressed PCNP was related with increased/ decreased expression of both intrinsic and extrinsic apoptotic executers, which factors are responsible for this dual behavior of PCNP? As reported (a) PCNP is ubiquitinated by NIRF which is up regulated during tumor,^22^ (b) ubiquitin is involved in membrane trafficking and (c) the involvement of PCNP in apoptosis through DNA damage response and in chromatin dynamics via STAT phosphorylation,^72^ are important factors which evoke the PCNP involvement in cell cycle regulation, transcription and apoptosis. In conclusion, we recommend to explore PCNP in above mentioned expects at molecular level to understand its role in cancer progression and to develop cancer therapeutics. The insights into the export mechanism of PCNP will improve our understanding about apoptosis. The biological half‐life of short‐lived PCNP can be improved if we would have enough information about ubiquitination of PCNP.

Presently, PCNP is under initial investigations for its potential for ubiquitination and tumor inhibition with few published and some unpublished reports. However due consideration is required to investigate its regulatory and transcriptional values. Looking forward, being nuclear resident with PEST motif, further findings about PCNP in different cellular conditions such as the cell cycle and signaling will devise new therapeutic strategies.

## CONFLICT OF INTEREST

All the authors declare no conflict of interest.

## Data Availability

Not applicable.
